# An Era of Digital Healthcare—A Comprehensive Review of Sensor Technologies and Telehealth Advancements in Chronic Heart Failure Management

**DOI:** 10.3390/s24082546

**Published:** 2024-04-16

**Authors:** Tejaswini Manavi, Haroon Zafar, Faisal Sharif

**Affiliations:** 1 Cardiovascular Translational Research & Innovation Centre, University of Galway, H91 TK33 Galway, Ireland; t.manavi1@universityofgalway.ie (T.M.); faisal.sharif@universityofgalway.ie (F.S.); 2 Lambe Institute for Translational Research, School of Medicine, University of Galway, H91 TK33 Galway, Ireland; 3 College of Science and Engineering, University of Galway, H91 TK33 Galway, Ireland; 4 Department of Cardiology, University Hospital Galway, H91 YR71 Galway, Ireland

**Keywords:** heart failure, haemodynamic monitoring, remote monitoring, remote patient management, sensors, telehealth, telemonitoring, invasive, non-invasive

## Abstract

Heart failure (HF) is a multi-faceted, complex clinical syndrome characterized by significant morbidity, high mortality rate, reduced quality of life, and rapidly increasing healthcare costs. A larger proportion of these costs comprise both ambulatory and emergency department visits, as well as hospital admissions. Despite the methods used by telehealth (TH) to improve self-care and quality of life, patient outcomes remain poor. HF management is associated with numerous challenges, such as conflicting evidence from clinical trials, heterogeneity of TH devices, variability in patient inclusion and exclusion criteria, and discrepancies between healthcare systems. A growing body of evidence suggests there is an unmet need for increased individualization of in-hospital management, continuous remote monitoring of patients pre and post-hospital admission, and continuation of treatment post-discharge in order to reduce re-hospitalizations and improve long-term outcomes. This review summarizes the current state-of-the-art for HF and associated novel technologies and advancements in the most frequently used types of TH (implantable sensors), categorizing devices in their preclinical and clinical stage, bench-to-bedside implementation challenges, and future perspectives on remote HF management to improve long-term outcomes of HF patients. The Review also highlights recent advancements in non-invasive remote monitoring technologies demonstrated by a few pilot observational prospective cohort studies.

## 1. Introduction

HF is a multi-faceted, complex clinical syndrome characterized by significant morbidity, mortality, and reduced quality of life [1]. It remains the leading cause of death in the United States, with 695,000 deaths in 2021 reported by the Centers for Disease Control and Prevention [2]. As per the Heart Disease and Stroke Statistics 2023, cardiovascular disease (CVD) accounted for 928,741 deaths in the year 2020, with 85% accounting for HF and stroke [3].

The prevalence of HF has increased over the last few decades due to the aging population, co-morbidities (diabetes, hypertension, chronic obstructive pulmonary disease [COPD]), and improved survival rates following effective therapies [4,5]. It is also the most common cause of CVD-related hospitalization in patients older than 60 years of age [5]. In Ireland, the total cost of HF was estimated at approximately €660 million in 2012, and expenditure per HF admission was estimated to be more than €10,000 [6]. After a decade, the annual record of HF hospital admissions was approximately 5800 in 2022, and the outpatient department (OPD) waiting times varied between 6 and 9 months on average. This creates a significant barrier to managing HF in primary care [7].

In patients ≥65 years of age, HF co-exists with other comorbidities that makes HF diagnosis challenging [8]. Despite HF diagnosis, admission rates account for 10% of patients attending the emergency department (ED) with worsening symptoms within a year [9]. Moreover, HF is also a major cause of recurrent hospitalizations due to poor prognosis [10] and low patient adherence to guideline-directed medical treatments and lifestyle changes [11,12]. 

Over the past few decades, advancements in remote patient monitoring (RPM) technologies have paved the way for proactive management of HF. Early detection of worsening HF symptoms enables timely pharmacological intervention, which can reduce or prevent hospitalizations [13]. RPM is a form of telemedicine, where the provision of care is available to patients at-home from their care provider at a different location using either information technology or artificial intelligence [14]. 

Although the terms RPM, Telehealth (TH), and Telemonitoring are used interchangeably, they have distinct definitions. RPM is a subset of Telehealth. TH refers to the medical practitioners’ use of information and communications technology to deliver healthcare services, facilitating the exchange of reliable information for diagnosis, treatment, and disease prevention. It extends to monitoring, assessment, and ongoing education for healthcare professionals, all aimed at promoting individual and community health, especially in situations where distance is a critical factor. Telemedicine is another form of TH that includes patient counseling, case management, and patient supervision by health professionals. The remote exchange of physiological data between a patient at home and a healthcare professional at the hospital to aid in diagnosis is referred to as Telemonitoring. It uses service systems and devices to remotely collect and transmit vital signs to a monitoring platform for interpretation or review [15]. 

RPM has evolved over time with the introduction of various methods of remote patient care or management. RPM methods can be categorized as either asynchronous (non-simultaneous, stored data) or synchronous (simultaneous, real-time data) and depend on periods of information transfer. Both these methods have been used in patients with chronic HF, either individually or in combination [16]. According to the existing literature, TH methods for HF patients [17,18,19,20] mainly involve the following:

- Telephone support systems
- Implants (invasive): Cardiovascular electronic devices (ICDs, CRT-Ds), hemodynamic sensors (filling pressures such as right ventricular pressure, pulmonary artery pressure, left atrial pressure, central venous pressure, interstitial fluid pressure) for remote intra cardiac monitoring.
- Digital platforms (non-invasive): Commercial devices to measure vital signs (blood pressure, daily weight, heart rate, oxygen saturation, ECG) and assessment of HF symptoms (app-based surveys, medication adherence).
- Wearable technologies (non-invasive): smart watches, patches, or textiles to monitor body temperature, blood glucose levels, ECG, and body posture.

These approaches are used individually or in combination to provide multidisciplinary and integrated care to HF patients, which is the current gold standard for HF management. Although the experience with RPM was published in the mid-1990s, we are still decades away from its widespread adoption and implementation into daily clinical practice [21,22]. The early phases focused on non-invasive forms of remote monitoring, but the results were largely inconsistent. A Cochrane review (2015) evaluated 25 structured telephone support (STS) studies with 9332 patients and 18 non-invasive telemonitoring studies with 3860 patients and compared them to the usual standard of care. Both the STS and non-invasive remote monitoring showed a beneficial effect in reducing all-cause mortality and HF hospitalizations. However, neither of them demonstrated a reduction in the risk of all-cause hospitalizations and quality of life. The limitations of these studies include different inclusion and exclusion criteria between both the groups, heterogeneity of compared data, mixed interventions ranging from telephone calls only to vitals monitoring, and complex telemonitoring management strategies, making it challenging to conclude what intervention or therapy drives the effect [22]. The effects of RPM on morbidity and mortality have also been studied in some non-invasive telemonitoring randomized controlled trials. TIM HF2 trial is the first non-invasive RPM intervention trial that reported morbidity and mortality outcomes up to 1 year after the termination of RPM intervention. The results were promising and showed modest benefits on cardiovascular hospitalizations and all-cause mortality. Non-invasive remote monitoring can have potentially significant benefits in the management of HF patients, specifically lower-risk (NYHA I/II) patients. This can be ensured by its simple and holistic approach combined with patient education, continuous 24/7 telemonitoring strategy, and profiling of the patient necessary to assess their healthcare needs and preferences in order to achieve the appropriate clinical response after enrolment into the study [23,24]. 

The shift to invasive forms of remote HF monitoring allows for more optimized care with access to multiple physiologic variables. Examples include cardiac implantable electronic devices (CIEDs) to measure tachyarrhythmia burden, heart rate variability, percentage of biventricular pacing, autonomic function, and intrathoracic impedance, and dedicated sensors to measure intra-cardiac filling pressures (pulmonary artery pressure, left atrial pressure, central venous pressure, interstitial fluid pressure) [25]. Several studies, such as PARTNERS-HF, Triage-HF, and MultiSENSE, have demonstrated the efficacy of the transthoracic impedance measured by CIEDs to predict HF hospitalizations (HFH) [26,27,28]. The PARTNERS-HF is the largest prospective study demonstrating the feasibility of combined HF device diagnostics to risk-stratify patients over specific periods. PARTNERS-HF study (*n* = 694) measured several other parameters along with the impedance (Fluid index, OptiVol) to create a monthly score that predicted HFH and observed a 5.5-fold increased risk of HFH within a month. However, such studies may be limited by the specificity of the device models or biased due to trials not being blinded and non-randomized trials with uncertainty over whether the clinical interventions based on HF device diagnostic lead to an improvement in the outcomes [28]. 

More recently, the scope has been shifting towards wireless hemodynamic sensors that measure intra-cardiac filling pressures or surrogates of these pressures. Hemodynamic congestion precedes clinical congestion in days or even weeks [29]. The advent of small pressure sensors placed in the vasculature can help detect early signs of congestion, and the hemodynamic data can thus serve as better markers for these filling pressures.

This narrative review provides an overview of recent medical technological innovations and advancements in the field of remote HF management, specifically the hemodynamic sensors (invasive) and digital platforms (non-invasive). It also highlights the ongoing studies (preclinical, clinical trials, pilot, or observational studies) and future perspectives on using RPM techniques that impact HF care globally. 

## 2. Sensor-Based HF Monitoring

It is already known that sensors are most commonly used in medical applications, embedded into smartphones and wearable devices for detecting, recording, and reviewing physiologic parameters (patient’s vital signs). In addition, they are increasingly incorporated into implantable devices for CVD prevention, more specifically HF, that involves home monitoring of hemodynamic parameters. The non-invasive or wearable sensor types include accelerometers (physical activity, walking gait), infrared photo-detectors (body temperature, heat flux, and heart rate), glucometers, and ultrasound patches (cardiac imaging) [30]. Implantable sensors are the newly emerging and arguably the most important means for continuous HF monitoring. Examples include electrophysiologic sensors (intrathoracic/intracardiac impedance [ICDs, CRT-Ds], heart rate variability, physical activity, minute ventilation), hemodynamic sensors (right ventricular pressure, left atrial pressure, pulmonary artery pressure, central venous pressure) biochemical sensors (mixed venous oxygen saturation), and arrhythmia sensors (atrial arrhythmias [CRT-Ds]) [31].

### 2.1. Haemodynamic Sensors in Clinical Practice

#### 2.1.1. Left-Atrial Pressure (LAP) Monitoring

One of the ideal parameters to target HF therapy is the pulmonary capillary wedge pressure (PCWP) or the left atrial pressure, which can accurately reflect changes in blood volume and left ventricular end-diastolic pressure (LVEDP) [32]. Several clinical trials have successfully demonstrated measurements of LAP and its clinical implications in reducing device-related complications, HF hospitalizations, and all-cause mortality. LAPTOP-HF trial is a multi-center, prospective, randomized clinical trial with a trans-septal sensor HeartPOD implanted in the atrial septum of a certain cohort of HF patients (*n* = 486, NYHA class 3, history of HF hospital admissions within the past year). This study revealed that the patients followed up by medical therapeutic interventions directed by LAP measurements showed a 41% reduction in HF hospitalizations at 12 months. Patients used a handheld device to measure sensor readings twice daily and communicated wirelessly with their healthcare providers for any guideline-directed medical therapy (GDMT) adjustments or lifestyle recommendations. However, the data safety and monitoring board terminated this study due to an excess of transseptal-related procedural complications [33]. In addition, the preliminary results of this trial presented at the HF Society of America (2016) demonstrated negative results with no reduction in combined HF recurrent hospitalizations and therapeutic complications [34]. The HeartPOD sensor is capable of measuring LAP waveforms, atrial ECG, and body temperature. The safety, accuracy, and feasibility of ambulatory LAP measurements were demonstrated in a small clinical study involving eight patients [35]. In another study (HOMEOSTASIS trial), HeartPOD was implanted in forty patients with NYHA class III/IV, where LAP measurements were used to optimize medication titrations during the 3-month observation period. Outcomes showed a significant decrease in the mean LAP measurements between the observatory and stability period (*p* = 0.003). The proportion of cardiovascular medication titrations increased significantly during the stability period, resulting in patients receiving optimized doses of angiotensin-converting-enzyme inhibitors/angiotensin receptor blockers, beta-blockers (up-titrations), and loop diuretics (down-titrations) [36]. The device was successfully implanted in all forty patients. Device failure or malfunction was reported in four patients [36], and five of them underwent percutaneous sensor lead extraction because of infection [37]. 

**Figure 1:**
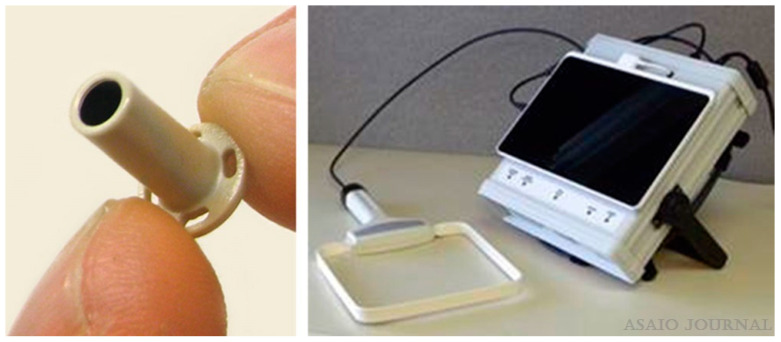
Titan^TM^ Pressure Sensor (left panel). The sensing element is located at the distal end, whereas the proximal end has four holes for fixation with sutures. The recording unit includes an antenna and a tablet phone for home monitoring (right panel) [38]. Copyright 2016, with permission from Elsevier.

Another study involving 40 HF patients demonstrated the safety and feasibility of measuring LAP using the Titan^TM^ pressure sensor (Figure 1) [38]. The sensor was implanted in thirty-one patients scheduled for open-heart surgery and nine scheduled for transcatheter aortic valve replacement (TAVR). This first-in-man study wirelessly measured LAP against reference pressure values such as fluid-filled and Millar catheters (Millar Instruments, Inc., Houston, TX, USA). The sensor did not require calibration and showed a good correlation with the reference pressure values. The control group involved showed no adverse clinical events or complications. During the pre-clinical stage, the sensor was tested long-term (27 weeks approx.) on more than 60 animals and demonstrated favorable results in terms of biocompatibility, thrombogenicity, and feasibility. This study was also the first one to demonstrate the feasibility of implantation in all four chambers of the heart, aortic and pulmonary arteries. It also emphasized the potential of home monitoring to detect early signs of increasing filling pressures and the importance of enabling therapeutic interventions before the occurrence or worsening of any symptoms [38]. Although this technology was reported to be safe and accurate over a certain time point, it did not demonstrate long-term clinical benefits. A case study reported the functioning of the Titan^TM^ pressure sensor in four patients with a left ventricular assist device (LVAD) that allows for precise management of intravascular volume and understanding correlations between LAP, pump speed, and left atrial (LA) and left ventricular (LV) size [39]. Outcomes indicated significant correlations between these parameters and the potential of LAP measurements in regulating optimal pump speed in patients with LVAD. An interesting aspect of this study is that the hemodynamic sensor for LAP monitoring was integrated with LVAD technology, making it a clinically beneficial strategy for HF monitoring. However, the limitations include a smaller sample size (*n* = 4) and a shorter follow-up time (36–180 days). Out of the four patients, one patient died after 36 days in ICU due to right-sided HF and multi-organ failure. An autopsy report showed the LAP sensor was intact, with no adverse reactions in the area surrounding the sensor [39]. 

**Figure 2:**
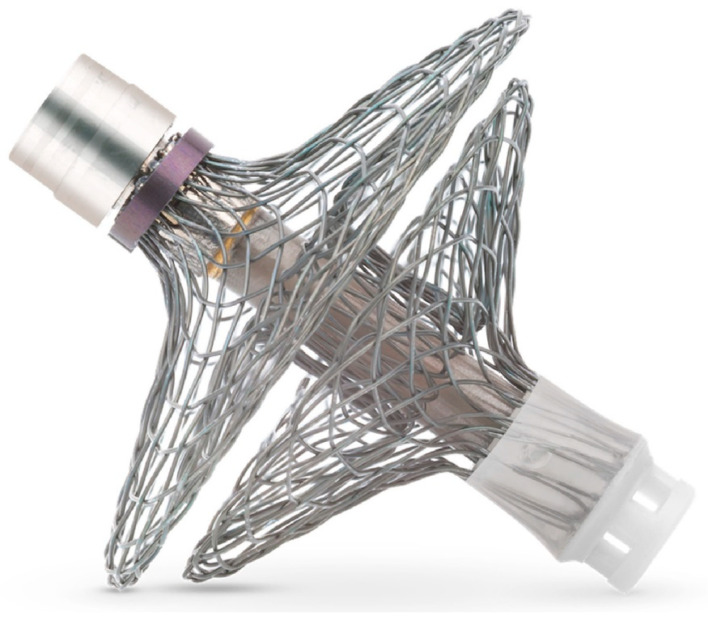
V-LAP System (Vectorious Medical Technologies) is a wireless intracardiac pressure sensor placed in the interatrial septum via transseptal access [43], Creative Common CC BY License.

Moreover, an increase in LAP is an indicator of many HF conditions, such as left ventricular hypertrophy, mitral valve regurgitation, and cardiac ischemia, which implies a higher clinical value of LAP monitoring in HF patients [40,41,42]. A clinical trial demonstrated the importance of remote LAP measurements in guiding HF patient management through an advanced version of LAP monitoring, V-LAP^TM^, in the VECTOR-HF trial [43] (Figure 2). The safety, usability, and performance were assessed in 30 HF patients followed for 3-months. Post implantation, a Swan–Ganz catheter was inserted to measure the mean PCWP invasively, and this was correlated with simultaneous mean LAP measurements obtained from the V-LAP sensor. The study demonstrated a good correlation between invasive PCWP and its surrogate LAP and significant improvements in clinical symptoms such as NYHA class (60% of patients improved from Class 3 to 2) and freedom from major adverse cardiac and neurological events (97% at 3 months). The next-generation V-LAP^TM^ monitoring system is a miniature, percutaneous, wireless, and leadless pressure sensor implanted permanently in the atrial septum using a transseptal approach. The system comprises four parts: a leadless LAP sensor with a low profile design and a novel drift compensatory mechanism, a dedicated delivery system with a repositioning mechanism, an external reader device in the form of a wearable belt that remotely powers the sensor and a secure web-based database available to clinicians for review. The implant features a hermetically sealed body housing sensing elements and a nitinol braided double disc-anchor. The discs are deployed on both sides of the interatrial septum, ensuring firm positioning post-implantation while the sealed body crosses the septum. The wearable belt can be placed over the clothing around the chest for 1 to 3 min to conduct daily measurements. The pre-clinical testing of the V-LAP^TM^ monitoring system demonstrated the safety, accuracy, and efficacy of measurements with the wearable belt at depths of up to 30 cm [33,43]. Although the experiments with the V-LAP^TM^ device were a big success, the limitations include indirect measurements of LAP, i.e., PCWP, and the requirement to validate the accuracy of measurements beyond hypervolemic and hypertensive conditions [43,44]. 

#### 2.1.2. Right-Ventricular Pressure (RVP) Monitoring

**Figure 3:**
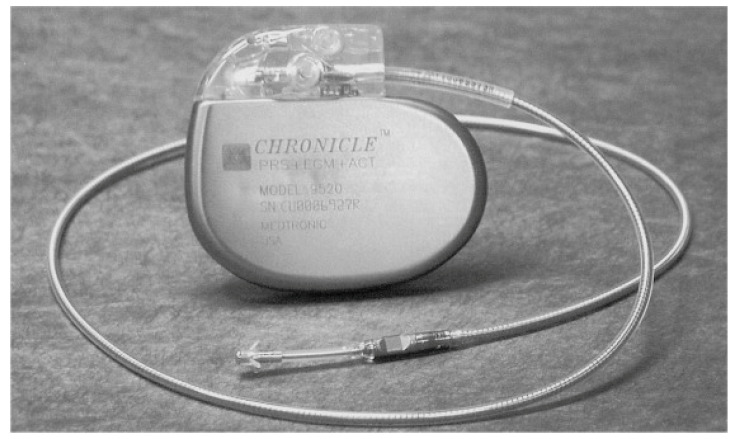
The Chronicle IHM System features a single right ventricular lead housing a pressure transducer placed 2.8 cm away from the tined distal tip electrode [45], Copyright 2002, with permission from Elsevier.

The Chronicle Right Ventricular Pressure Monitoring System shows promising results with a good correlation to pulmonary artery diastolic pressure, the main hemodynamic variable responsible for HF-related events [45,46]. A study tested the Chronicle Implantable Haemodynamic Monitoring (IHM) System (Medtronic, Inc., Minneapolis, Minnesota) (Figure 3) in 32 HF patients, which recorded RV systolic and diastolic pressures and its derivatives and heart rate [47]. The pressures increased gradually, and the increase was evident approximately 4 days before the exacerbation. Pressure-guided therapy in response to elevated pressures enabled a significant 57% reduction (*p* < 0.01) in HF admission rates [47]. In a follow-up study, known as COMPASS-HF (Chronicle Offers Management to Patients with Advanced Signs and Symptoms of HF) [48], HF patients (*n* = 274) with NYHA class III and IV were randomized to Chronicle IHM group versus the control group. Patients in the NYHA class IV category required intravenous therapies due to resistance to oral diuretic agents, necessitating hospitalizations and preventing effective treatment of their elevated pressures. Therefore, the reduction in HF-hospitalizations and emergency department (ED) visits was non-significant. This study retrospectively analyzed the time to first hospitalization and observed a 36% reduction (*p* < 0.05) in the relative risk of HF hospitalizations in the treatment group. Only the NYHA class III patients benefited from such pressure-guided therapy. The number of decompensation events in NYHA functional class IV patients randomized to the treatment group was greater compared with the control group. The observation that elevated pressures precede the occurrence of HF symptoms and the necessity to modify therapy in response to these elevated pressures, even in the absence of signs and symptoms, is the rationale for the next generation of implantable devices [48]. 

A predefined subgroup analysis in the COMPASS-HF trial aimed to evaluate the Chronicle IHM device’s effectiveness in patients with HF preserved ejection fraction (HFpEF) of 50%. This analysis holds significant importance as it pertains to a substantial portion of congestive HF (CHF) patients who frequently experience hospital readmissions due to acute HF. Furthermore, there is a limited availability of guideline recommendations for HF therapy in this specific subgroup [49,50,51,52]. A total of 70 randomly assigned patients (*n* = 34 in the treatment group; *n* = 36 in the control group) were examined as part of the subgroup analysis. In line with the primary analysis, the reported 20% reduction in the overall rate of HF-related events did not reach statistical significance (*p* = 0.66). Similarly, the reduction in the relative risk of HF hospitalizations was also not statistically significant (29% reduction, *p* = 0.43). It is worth noting that, despite the pre-planned nature of the subgroup analysis, the sample size may have been insufficient to assess the efficacy outcomes. In total, there were nine complications related to the system and two complications related to the procedure. 

Developments in the arena of hemodynamic pulmonary artery pressure (PAP) monitoring have been evaluated, and new implantable sensors are on the horizon, focusing on real-time and longitudinal measurements of PAP rather than surrogates of such pressures. 

#### 2.1.3. Pulmonary Artery Pressure (PAP) Monitoring

Recent literature studies provide new evidence for PAP monitoring, demonstrating two options: the CardioMEMS HF System and the Cordella PA Sensor System. This section highlights the completed and ongoing clinical trials for remote PAP monitoring in both the EU and US settings with a future perspective on remote HF care. 

##### CardioMEMS HF System

**Figure 4:**
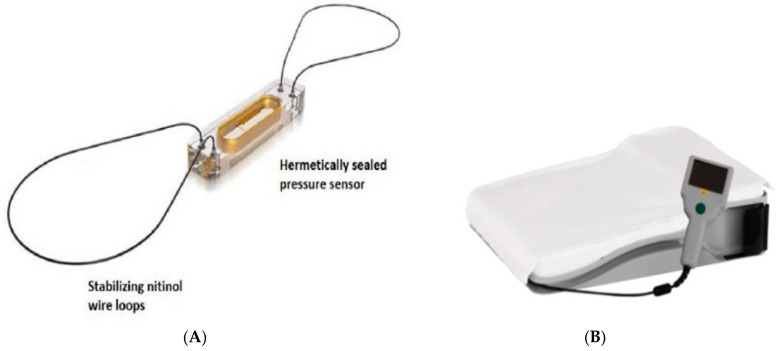
CardioMEMS System with (**A**) The Pulmonary Artery Pressure Sensor (Abbott) and (**B**) Electronic External Unit (pillow) for daily pressure readings, and ensures secure transmission of patient data to a cloud server for clinical assessment. Used with permission from Abbott Inc. (Abbott, Sylmar, CA, USA).

To date, the most remarkable breakthrough in the realm of implantable hemodynamic monitoring capabilities has been achieved through the introduction of a novel, wireless, and battery-less system for the direct measurement of PAP, known as CardioMEMS HF System (developed by Abbott, Sylmar, CA, USA) (Figure 4). This system consists of a compact sensor implanted within a branch of the pulmonary artery, enabling the daily measurement of systolic, diastolic, and mean PAP. Patients perform PAP measurements once a day, while the clinicians acknowledge pressure data through a secure cloud-based patient network. It is already known that an elevation in PAP occurs during the initial phases of cardiac decompensation well in advance of the manifestation of clinical signs and symptoms. Consequently, this provides clinicians with the opportunity to intervene at an early stage to forestall clinical decompensation and the ensuing hospital admissions. The procedural details of CardioMEMS have been elaborately described elsewhere [53,54]. In summary, the CardioMEMS HF system is an implanted sensor specifically designed for placement within the (left) pulmonary artery (Figure 4A). This sensor operates without a battery as it utilizes radio frequency energy transmitted from the measurement device employed for daily readings. Moreover, the sensor is entirely compatible with Implantable Cardioverter–Defibrillators (ICDs) and Cardiac Resynchronization Therapy (CRT) devices. It is externally powered by a patient electronics unit. Patients are advised to perform readings every day using a device in the form of a pillow that connects to the sensor (Figure 4B). Each reading lasts about half a minute and captures data on mean pulmonary artery pressure (mPAP), systolic PAP (sPAP), diastolic PAP (dPAP). Physicians can make informed treatment decisions by analyzing trends in mPAP and can establish customized targets and thresholds for warning signs and symptoms. Importantly, the CardioMEMS sensor is MRI-compatible and designed for lifelong durability. Patients are treated with anticoagulants post-sensor implantation. The sensor is designed for permanent placement as endothelialization occurs after adequate treatment with anticoagulants [53,54].

##### Clinical Evidence

The CHAMPION (CardioMEMS Heart Sensor Allows Monitoring of Pressure to Improve Outcomes in NYHA Class III HF Patients) trial [54] enrolled 550 patients with chronic HF (NYHA III) with clinical endpoints focusing on a reduced risk of HF hospitalization, or device and procedure-related complications. In this study, patients were randomly assigned to a treatment group, where clinicians relied on PAP-guided treatment decisions and a control group received only standard HF care (without PAP data). [53,55]. This study also looked at the frequency of medication changes between the treatment and control groups with a hypothesis that a higher frequency of medication changes based on PA pressures consequently reduces HF hospitalization rates. Patients with higher baseline PA pressures received more frequent medication therapies, so a reduction in their PA pressures, in turn, reduces the risk of HF hospitalization. The treatment group (2468) had significantly higher medication changes than the control group (1061) (*p* < 0.0001). The treatment group received more medication titrations, specifically the diuretics. These were mostly down-titrations in the diuretic drug doses and occurred more frequently in the treatment group compared with the control. Additionally, significant increases in the other class of GDMT drug doses (ACEi/ARB, beta blocker, and MRA) were observed between baseline and 6 months in the treatment group compared with the control. [56]. At 6 and 15 months, the HF-related hospitalizations reduced by 28% and 37%, respectively, in the treatment group as with the control group (hazard ratio (HR) 0.72, 95% CI 0.60–0.85 and HR 0.63, 95% CI 0.52–0.7, respectively). A total of eight device-related complications and seven procedural-related complications were reported. Following these outcomes, the device/technique was considered to be safe, and FDA approval was established in 2014 [53,55]. 

A second randomized clinical trial known as GUIDE-HF (Hemodynamic-guided management of HF) [57], similar to the CHAMPION trials, was conducted at multiple centers in the US and Canada. This study included a total of 1000 patients from a broad HF category of NYHA II-IV with either a recent HF hospitalization or elevated natriuretic peptides. As seen in the CHAMPION trial, patients in the GUIDE-HF study were identified as a treatment group (PAP-based treatments) and a control group (clinicians blinded to PAP measurements). Primary endpoints focused on evaluating both all-cause mortality and the occurrence of total HF-related events. There were no significant differences in the primary (HR 0.88, 95% CI 0.74–1.05) or secondary endpoint (HR 0.85, 95% CI 0.70–1.03). However, due to COVID-19, a pre-sensitivity analysis was performed, which showed a significant reduction in the risk of all-cause mortality in the treatment group (HR 0.81, 95% CI 0.66–1.00). This was attributed to a reduction in the rate of HF events (HR 0.76, 95% CI 0.61–0.95). During the COVID-19 pandemic, there was a significant reduction of 21% in the HF event rate within the control group, whereas the event rate in the treatment group remained unchanged. Moreover, the frequency of medication changes was higher in the treatment group compared with the control [57]. 

Clinical evidence for CardioMEMS is limited to the U.S., highlighting a gap in European clinical trial data. However, non-randomized observational studies conducted in European settings have yielded promising results in reducing HFH. The MEMS-HF study started in Germany in 2020 and was subsequently extended to include other countries such as Ireland and the Netherlands [58]. The patient inclusion criteria were similar to the clinical trials discussed above, with additional endpoints being the patient quality of life assessed by the Kansas City Cardiomyopathy Questionnaire (KCCQ). During this trial, 234 patients received the CardioMEMS implant. A significant reduction in HFH (66%) was observed at 12 months (HR 0.34, 95% CI 0.26–0.44), which exceeded the reductions observed in the CHAMPION trial. Moreover, reductions in mean PAP by 3.4 mmHg and 5.5 mmHg were reported at 6 and 12 months, respectively (*p* < 0.0001). Improvements in patient quality of life (KCCQ, Patient Health Questionnaire depression module, ED-5D-5 L questionnaire) were observed, with no deterioration at 12 months [58]. 

Despite the successful demonstration of these trials, the limitations include the nature of the study (observational, non-randomized), the absence of a control group (patients served as their own historical control), and the study design, which is susceptible to significant biases, thus limiting the ability to determine the actual effect of such studies. 

##### Safety & Clinical Efficacy

An observational open-label Post Approval Study (PAS) validated the safety and effectiveness of CardioMEMS in NYHA class III HF patients (*n* = 1200) with a history of HFH one year prior to enrolment [59]. A significant reduction in the rate of HFH at 12 months was observed compared with one year before implant (HR 0.43, 95% CI, 0.39–0.47). Recently published results for the 2-year study show that HFH rates continued to decrease over time. By the end of the first year, the HFH rates dropped from 1.25 to 0.54, and by the end of the Recently published 2-year results demonstrated a continued reduction in HFH rates from 1.25 to 0.54 at 1 year and further to 0.37 in the second year (*p* < 0.0001 for both 1 and 2-year follow-ups). In the sub analysis of 710 patients completing 24 months of follow-up, a similar pattern was observed. The primary interventions in PAS involved adjustments to loop diuretics and the temporary addition of thiazide diuretics. Researchers suggested that the benefits of PA pressure-guided management were likely due to the optimization of diuretic therapy. Additionally, it is worth noting that a significant proportion of patients with HF with Reduced Ejection Fraction (HFrEF) were already on GDMT at baseline [60]. 

The extensive Medicare database is the primary source that provides real-world evidence of CardioMEMS studies [61,62]. In the first study (*n* = 1114), the HFH rate 12 months post-implantation was 34% lower than the 12 months before (HR 0.66, 96% CI 0.57–0.76) [62]. In the second study, 1087 CardioMEMS patients were matched with 1087 controls. During the post-12-month period, the CardioMEMS group had a lower HFH rate (HR 0.76, 95% CI 0.65–0.89) [61]. Additionally, a study analyzed the Nationwide Readmissions Database (NRD) to identify acute HF hospitalizations over a period of five years. Patients were categorized based on who received the CardioMEMS implant and who did not. In both matched and unmatched analyses, patients with CardioMEMS had significantly lower readmission rates at specific intervals (30, 90, and 180 days) compared with those without [63].

Another study that assessed the safety, effectiveness, and feasibility of CardioMEMS is the COAST (CardioMEMS HF System Post Market Study) study [64]. This prospective multicentre open-label study enrolled 130 patients from the UK, Europe, and Australia. As mentioned previously, the inclusion criteria for patients are NYHA class 3 and at least one HFH in the past year from enrolment. In 2021, a subset of the UK study results with patients enrolled prior to the COVID-19 pandemic were published. The main safety endpoints (device-related serious complications, sensor failure, and HFH rate two years post-implantation) were in line with earlier observational studies. There was an 82% reduction in the risk of HFH rate post-implantation. Additionally, PAP showed a significant decrease during follow-up. Similar to prior studies, the majority of the medication changes consisted of adjustments to diuretics. The limitations of this study are the same as the MEMS-HF study, which is the non-randomized design and a missing comparator arm [64]. 

A pilot study in the Netherlands called HEMOVADS assessed the safety and feasibility of CardioMEMS in patients with a left ventricular assist device (LVAD). The hypothesis was based on the notion that optimizing hemodynamics might lower the risk of renal and right ventricular (RV) failure, help manage fluid levels after LVAD implantation, and offer personalized outpatient care through remote monitoring [65]. The indication approach used for CardioMEMS in this study is different from other studies mentioned earlier. In conclusion, this pilot study demonstrated the safety and feasibility of the approach in LVAD patients [65,66,67]. 

The MONITOR-HF study, conducted across 25 centers in the Netherlands [68], employed an open-label, randomized design to investigate the impact of hemodynamic monitoring using the CardioMEMS HF system (Abbott Laboratories, Abbott Park, IL, USA) on patients with chronic HF. The inclusion criteria were well-defined, encompassing individuals with NYHA class III HF and a history of previous HF hospitalization, regardless of ejection fraction. Participants were assigned randomly to either undergo hemodynamic monitoring using the CardioMEMS-HF system or receive standard care. This study stands out due to its distinctive approach, with the primary focus on assessing the quality of life using the Kansas City Cardiomyopathy Questionnaire. This unique choice enables researchers to establish a connection between hemodynamics and the overall quality of life, providing valuable insights into the interplay between these factors. Findings revealed a significant improvement in the CardioMEMS-HF group compared with standard care. The responder analysis further supported these findings, indicating a higher odds ratio of improvement and a lower odds ratio of deterioration in the CardioMEMS-HF group. Notably, the study reported a high level of freedom from device-related (97.7%) or system-related (98.8%) complications, enhancing the safety profile of the intervention. Overall, the MONITOR-HF study presents compelling evidence for the efficacy and safety of hemodynamic monitoring using the CardioMEMS-HF system in managing chronic HF [68]. 

As of now, CardioMEMS is the sole PA pressure sensor incorporated into routine clinical HF care, having obtained both FDA approval and the CE mark. On the other hand, the Cordella Pulmonary Artery Pressure Sensor System (Endotronix, Inc., Lisle, IL, USA) serves as an alternate diagnostic modality with similar capabilities in remotely monitoring PA pressures. A unique feature of this system is the combined non-invasive component known as the Cordella HF System (CHFS), which measures additional vital parameters such as blood pressure, heart rate, oxygen saturation, and weight. The combination of both invasive and non-invasive elements, featuring an easily navigable patient–health care provider interface, has not been assessed previously. The FDA has approved the Cordella^TM^ PA sensor for Investigational Device Exemption (IDE) for a subsequent multicenter study, potentially broadening the application of the sensor system to a larger population of HF patients (NYHA II). A premarket approval (PMA) was submitted in early January 2024 following the completion of the enrolment for the PROACTIVE-HF trial. 

##### Cordella HF System

**Figure 5:**
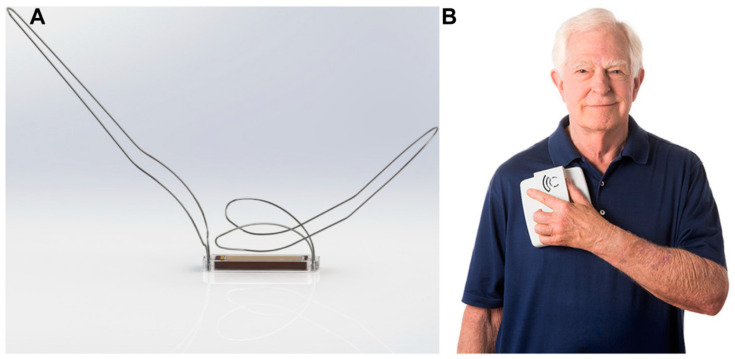
Cordella^TM^ Pulmonary Artery Pressure Sensor (Endotronix Inc., Lisle, IL, USA) (**A**); Handheld Reader that powers and interrogates the sensor and allows readings to be captured in both seated and supine positions (**B**) [69,70], Creative Commons CC BY License.

Cordella PAP System (Figure 5) by Endotronix, Inc. (Lisle, IL, USA) is currently undergoing two clinical trials [69,70]. The first one is an open-label, small-scale multicenter trial known as SIRONA that aimed to investigate the feasibility, safety, and accuracy of the Cordella^TM^ PAP sensor in 15 NYHA III patients. The inclusion criteria are similar to the CardioMEMS studies that involve NYHA III patients with a history of at least one HFH in the past 12 months. SIRONA trial demonstrated successful results in all fifteen patients; however, four cases (27%) of adverse events related to the procedure were reported, including one case each of sensor migration and transient complete heart blockage and two cases of hemoptysis. Notably, no complications related to the device system or sensor failure occurred within the initial 90 days post-implantation. Subsequent right heart catheterization at 90 days demonstrated a favorable correlation between invasively measured pressures and PA pressure, as measured by the Cordella^TM^ Sensor. The primary efficacy endpoint, which involved achieving a mean PA pressure at 90 days, was successfully met in all patients except one, resulting in a cohort difference of 2.7 mmHg. Safety considerations led to one patient not undergoing the 90-day right heart catheterization. Notably, the average patient compliance to daily measurements, i.e., the transmission of vital signs through CHFS and readings from the PA pressure sensor, were recorded as 99%, respectively [69]. 

The second study is a European CE Mark trial known as SIRONA 2 (ClinicalTrials.gov identifier: NCT04012944) that investigated the safety and efficacy of the Cordella PAP sensor [70]. A unique feature of this study is the inclusion of CHFS components (custom-app and commercial non-invasive devices to measure blood pressure, heart rate, oxygen saturation, and weight), enabling comprehensive management of NYHA III HF patients. The endpoints were categorized as primary efficacy endpoint that assessed the accuracy of mean PAP measured by the Cordella PA sensor versus standard right heart catheterization (RHC) at 3 months, and primary safety endpoint that determined freedom from adverse events associated with the Cordella PA sensor throughout 1-month post-implantation. The Cordella PA sensor was successfully implanted in 70 patients. There was a good correlation between the Cordella PA sensor and RHC for mean PAP, falling within the ±4.0 mmHg range (*p* = 0.003). Results demonstrated an excellent safety profile of the Cordella PA sensor, with 98.6% freedom from device-related complications (invasive treatments, device removal, or death). No cases of device failure were reported. Patients were highly compliant (94%) to the daily transmission of measurements for both PAP and vital signs [70]. 

Another similar trial, the PROACTIVE-HF study (NCT04089059) [71], was designed to investigate the replicability of the positive clinical outcomes observed in prior HF (HF) management trials guided by pulmonary artery pressure (PAP). Specifically, the study aimed to assess if similar favorable results could be achieved using the Cordella PA sensor system in diverse healthcare settings across the U.S. and Europe. PROACTIVE-HF trial is a prospective, multicenter, randomized, single-blinded study that aims to evaluate the safety and efficacy of the Cordella PA sensor. Both the randomized patient groups underwent implantation of the Cordella PA sensor. Patients were then categorized into a treatment arm and a control arm. In the treatment arm, HF treatment will be guided by monitoring of PAP, while the control arm will receive treatment based on GDMT and vital signs measured by CHFS alone. Patients in the control arm will receive PAP-guided HF management after 12 months. Changes in PAP measurements in both patient groups, as well as primary outcomes such as HFH, mortality, emergency department/outpatient intravenous diuretic visits, and device/system-related complications, will be reported at 12 months [71]. 

#### 2.1.4. Inferior Vena Cava (IVC) Area Monitoring

##### Foundry Innovation and Research 1 Ltd. (Fire1) System

**Figure 6:**
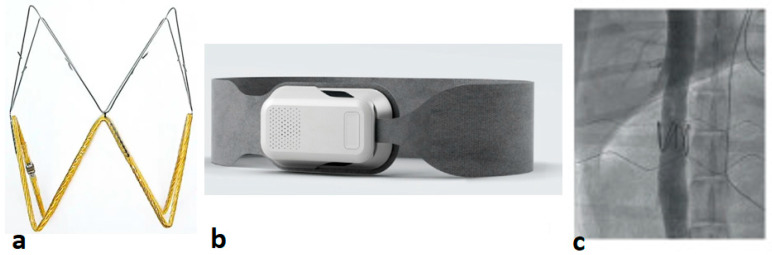
Fire1 (Foundry Innovation and Research 1 Ltd.) device measures IVC area and changes over time; (**a**) IVC area sensor; (**b**) Detection unit with a belt to energize the sensor; (**c**) Representation of Fire1 sensor implantation in a sheep model [72,73], Creative Commons CC BY License.

The Fire1 (Foundry Innovation and Research 1 Ltd., Dublin, Ireland) device is an IVC sensor that measures the area of IVC and its dynamic changes. The sensor is an electromagnetic resonator consisting of a coil of wire and a capacitor (Figure 6a), and it measures the cross-sectional area of the IVC using radiofrequency energy. The recording unit consists of a belt that energizes the implanted sensor and measures the resonant frequency. This is directly proportional to the area of the sensor (Figure 6b). The Fire1 sensor is implanted within the IVC using a specific delivery system and a 16F catheter sheath. The first animal study with a Fire1 device (*n* = 9) demonstrated successful results without any potential complications such as device migration, fractures, or thrombus (Figure 6c) [72]. Validation experiments performed in vitro revealed that variations in the area of the IVC were more sensitive compared with the changes in both cardiac and pulmonary pressures. This was observed across various interventions, including volume infusion (*p* < 0.001), vasodilation, and cardiac dysfunction induced by nitroglycerin (*p* < 0.001) and rapid right ventricular pacing (*p* ≤ 0.02), respectively. Subsequent animal studies assessed the safety of the device and demonstrated an implantation success rate of 100%. Notably, these studies showed a significant change in the IVC area with gradual volume instillations in contrast to changes in right atrial pressure (RAP). The primary endpoint of this study was to evaluate the relative efficacy of IVC dimensions versus invasive measurements of filling pressures in determining intravascular volume expansion, volume redistribution, and worsening cardiac function. [72]. 

A second animal study (*n* = 20) by Fire1 aimed to assess the safety and performance of the IVC sensor under acute and chronic intravascular volume modulation. Twelve animals received the IVC sensor, two animals received a control device (IVC filter), and a group of six animals received blood and saline infusions to study responses to volume challenges. No significant differences in normalized IVC area were observed at similar volume states (55 ± 17% on day 0 and 62 ± 12% on day 120, *p* = 0.51). Chronic integration of the sensors showed a thin, re-endothelialized neointima with maintained sensitivity to infused volume. Normalized IVC area significantly changed from 25 ± 17% to 43 ± 11% (*p* = 0.007) with 300 mL infused, whereas RAP required 1200 mL of infused volume before a statistically significant change from 3.1 ± 2.6 mmHg to 7.5 ± 2.0 mm Hg (*p* = 0.02) [73]. 

The main findings of this study indicate that increases in the IVC area are notably more responsive to volume loading than RAP. This implies that intravascular volume expansion could be identified at an earlier phase of fluid accumulation using this method. This could have important clinical implications by allowing earlier detection of hypervolemia compared with pressure sensors, leading to prompt clinical intervention and positively affecting the outcomes of HF patients. Additionally, implanting the sensor in the IVC and its integration through endothelialization has shown no negative effect on long-term sensor performance in response to fluid accumulation [73]. Nevertheless, there are certain limitations to consider in both animal studies, including experiments with healthy sheep, the possibility of potential side effects in relation to the duration of experiments conducted on the same day, and ambiguity concerning the application of experimental findings to humans, especially with HF. In addition, changes in the IVC area do not solely represent changes in the total volume of blood. Factors such as venous capacitance, IVC compliance, and pressure gradients (pericardial pressure and pressure around the myocardium) influence volume changes to varying extents [72]. Consequently, it is essential to interpret these results as hypothesis-generating. Further investigation with chronic HF animal models and HF patients is warranted to analyze the potential of IVC monitoring in HF management comprehensively.

In February 2023, the first-in-human implantation of the Fire1 device took place in the United Kingdom as part of the FUTURE-HF (First-in-Human-Clinical Investigation of the FIRE1 System in HF Patients) trial. The study focused mainly on replicating the safety and efficacy observed in a group of HF patients [72]. 

### 2.2. Haemodynamic Sensors in Preclinical Practice

#### 2.2.1. Central Venous Pressure (CVP) Monitoring

CVP is a key metric for the diagnosis of congestion and has the potential to predict a wider range of HF conditions. It also serves as a surrogate for RAP. Management of congestion plays a vital role in HF treatment as it helps to prevent the worsening of symptoms or health deterioration, eventually resulting in death [74]. Clinical congestion represents a key target for therapy but is associated with poor prognosis. [53,75]. Future HF monitoring systems rely on venous sensing to track systemic congestion and detect changes in intravascular volume through real-time monitoring of CVP and IVC area [72,73,76,77]. 

**Figure 7:**
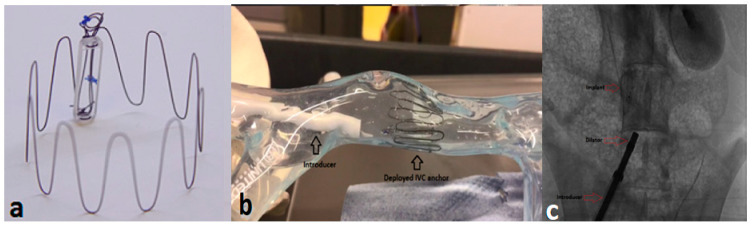
(**a**) IVC sensor for wireless CVP measurement; (**b**) Representation of IVC sensor deployment in a silicone bench-model of IVC; (**c**) Representation of IVC sensor implantation in a sheep model [76,78], Creative Commons CC BY License.

The preference for device implantation in a compliant vessel such as IVC has been outlined in a study involving a CT scan analysis to study the difference in IVC diameters in a group of patients with peripheral vascular disease (PVD) [77]. In addition, a study by Manavi et al. demonstrated the safety and efficacy of a novel IVC sensor (Figure 7a) for wireless measurement of CVP in an acute animal study. The IVC sensor implantation was carried out in two healthy sheep models at three different anatomical locations: IVC, superior vena cava (SVC), and the PA. These sensors were equipped with different anchor designs modified to meet specific anatomical requirements. A conventional PA sensor, commercially known as Cordella (Endotronix, Inc., Lisle, IL, USA), was implanted into both PA and SVC, while a sensor featuring a modified cylindrical anchoring system with stent-like struts was implanted in the IVC (Figure 7c). Calibration measurements of the sensors were performed against a Millar catheter reference (Millar Instruments, Inc., Houston, TX, USA). The effectiveness of central venous sensors in detecting pressure variations was evaluated by adjusting the fluid volume in animals. The study demonstrated successful implantation in both sheep, allowing simultaneous measurements to investigate correlations between PA and CVP. The implantation procedure was quick and took less than 15 min. A hand-held reader device was used to obtain multiple readings at each implant site under different conditions. Experiments revealed similarities in CVP measured at the IVC (6.49 mmHg) and SVC (6.14 mmHg) sites. As expected, PAP was higher (13–14 mmHg) than CVP. SVC waveforms showed clear beats and respiratory patters, while respiration was evident in IVC waveforms, although some beats were less distinct. Administration of additional saline to induce fluid overload indicated an increase in pressure in both SVC and IVC (3.7 and 2.7 mm Hg, respectively). In addition, a Bland Altman analysis illustrated a good correlation between sensor measurements and the Millar reference (Millar Instruments, Inc., Houston, TX, USA) [76].

According to recent literature studies focusing on hemodynamic HF monitoring, this is the only study that explored simultaneous measurements of pressures in three locations (IVC, SVC, and PA) in the same animal. Moreover, the study also shed light on the hypothesis that pressure in the superior (6.1 mmHg) and inferior (6.5 mmHg) veins is similar. Besides the animal implantation, this study is a first-of-its-kind to demonstrate the in vitro testing of the IVC sensor in a separate study using a bench model (Figure 7b) and computational fluid dynamics methods providing insights into the changes in blood flow due to the anchor and the resultant risks of medical device induced blood clotting and flow stagnation [78]. The clinical significance of real-time, non-invasive measurement of CVP focuses on the importance of proactive management of congestive HF with the aim to drive tailored diagnoses and treatment strategies. Introducing sensor technology into the vena cava offers a straightforward and minimally invasive solution for HF patients [78]. Nevertheless, further investigation in chronic animal studies with a larger sample size is required to assess simultaneous measurements in specific HF animal models and long-term accuracy compared with the gold-standard measurements (Millar Instruments, Inc., Houston, TX, USA). 

#### 2.2.2. Haemodynamic Sensing via a Vascular Electronic System (Wireless Arterial Stent)

While continuous hemodynamic monitoring has demonstrated enhanced patient outcomes, existing clinical devices are constrained by their bulky designs and inflexible materials, resulting in limited sensing capabilities. These devices are primarily suitable for monitoring pressure within the heart, abdominal aneurysms, and the PA, but they lack compatibility with other arteries [39,79,80]. However, strict requirements for implantation and operation have restricted the advancement of vascular electronics for arterial sensing. These requirements involve delivering sufficient wireless capabilities via a low-profile, miniaturized, and flexible system that can be securely placed within an artery and, at the same time, is compatible with existing minimally invasive catheter systems.

**Figure 8:**
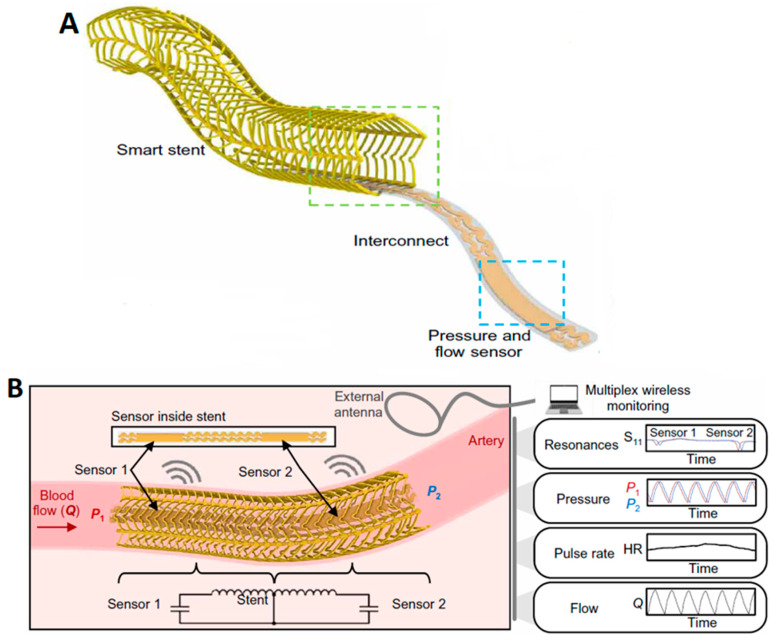
(**A**) Illustration of the electronic components of the Wireless Vascular Stent. (**B**) Representation of the sensor design and monitoring parameters (blood pressure, heart rate, and flow) [81].

A study by Herbert et al. introduced a wireless stent platform (Figure 8A) consisting of soft-printed sensors for measuring arterial pressure, pulse rate, and flow in real-time (Figure 8B) [81]. The design platform of the inductive stent is developed to enhance the wireless sensing features while retaining the essential characteristics of conventional stents. To provide a brief overview of the wireless sensing scheme, the integrated stent and sensors create inductor-capacitor (LC) circuits with a resonant frequency reliant on pressure. While only a sensor is required for monitoring the pressure, positioning a sensor at each end utilizes the stent, forming two LC circuits with distinct resonant frequencies. In order to capture both upstream and downstream pressures and detect the changes in flow rates, employing two sensors allows for monitoring of pressure gradient along the length of the stent. An external loop antenna and a vector network analyzer (VNA) wirelessly monitor the resonant frequency of each circuit using an S11 parameter (Figure 8B). This indicates that the wireless system facilitates real-time, simultaneous monitoring of pressure, pulse rate, and flow. 

The implantable, vascular electronic system with a wireless stent device illustrates multiplex sensing of hemodynamics across considerable distances within an artery model. The minimally invasive catheter implantation procedure of the vascular electronic stent was demonstrated in the narrow arteries of a rabbit model. Post-procedure, the implant site (right iliac artery) was extracted and examined to validate the functionality of the implanted wireless stent. Subsequent studies will investigate the endothelialization process, risk of re-stenosis, and stability of the vascular electronic stent in chronic animal models. 

Experiments conducted to assess the wireless performance of the vascular electronic system demonstrated a low power transfer efficiency attributed to the small size of the stent and a requirement to optimize the external reader system when coupled with the device. Hence, the scope further extends to the development of technical aspects, such as designing a new external reader for capturing signals from the device and modifying the stent by reducing the thickness of the sensor and struts. The goal is to improve efficiency, extend operating distance, and enhance reliability.

In conclusion, advancements in electronic designs, materials, and system integration present opportunities for remote sensing through implantable stent platforms, enabling a comprehensive approach to wireless hemodynamic monitoring [81].

Table 1 illustrates a summary of sensor technologies discussed above.

## 3. Non-Invasive Haemodynamic Monitoring

Non-invasive remote monitoring of HF has emerged as a new trend, aiming to deliver care from a distance by proactively managing signs and symptoms. These non-invasive methods incorporate mobile or app-based monitoring coupled with cloud technology, telephone-based interventions, or a combination of both, with the primary objective of minimizing hospitalizations and cardiovascular mortality and thereby enhancing patient quality of life. 

Several non-invasive methods for hemodynamic assessment are currently in development and are summarized in the following section. However, it is important to note that a comprehensive review of other clinically available non-invasive remote monitoring technologies (wearables, IoT) exceeds the scope of this review. Described below are some observational and randomized studies with a range of telehealth services in different clinical settings. Table 2 provides a summary of non-invasive telehealth technologies currently in clinical practice. 

### 3.1. Cordella HF System (CHFS)

**Figure 9:**
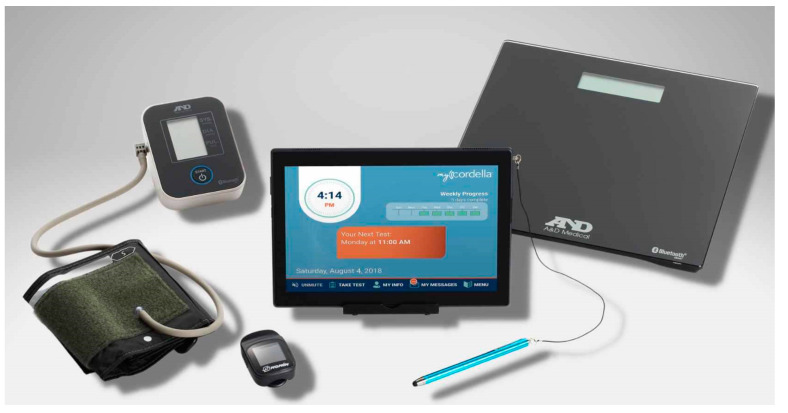
Cordella Heart Failure System (CHFS) (Endotronix, Inc. Lisle, IL, USA) with myCordella patient application and peripheral devices to measure blood pressure, heart rate, oxygen saturation, and weight [70,83]. Creative Commons CC BY License.

CHFS (Endotronix, Inc. Lisle, IL, USA) (Figure 9) is distinguished as the sole remote heart monitoring platform offering both ESC guideline recommended telemonitoring modalities, encompassing invasive (PAP) and non-invasive vital signs (BP, weight, HR, and SPO2), directly from patients’ home. Described below are a few studies that demonstrate positive patient outcomes utilizing only the non-invasive component of CHFS. 

A pilot study involving twenty-five HF patients at the University Hospital Galway, Ireland, explored the benefits of CHFS (Endotronix, Inc., Lisle, IL, USA), including real-time GDMT optimization, increased standard of care, facilitating a reduction in office-based visits and increased compliance to remote monitoring. The system utilizes Bluetooth-enabled devices for collecting vital signs such as blood pressure, pulse, oxygen saturation, and body weight, along with a tailored health questionnaire through a wireless tablet. This data is promptly accessible to healthcare providers via a cloud-based server [83]. An extended follow-up with 30 patients at 12 months of enrolment revealed excellent patient and clinic compliance of 92% and 94%, respectively. The clinicians utilized the cloud-system at-least twice weekly to acknowledge patient data and communicated GDMT optimizations or lifestyle adjustments via either a telephone call or cloud-based messaging. A significant reduction (54%) in HF hospitalizations was observed at 12-months (*p* < 0.05). However, there was no difference in the overall summary scores for the quality of life health questionnaire (KCCQ) from baseline through 6 months (53.7 ± 23.2 vs. 53.7 ± 21.3, *p* = 0.99). Additionally, an improvement in the patient NYHA class was observed at 6 and 12-month follow-ups, though this was not statistically significant [84]. 

Another study utilized CHFS in a private clinical setting in Texas, USA, with the primary endpoint to reduce HF Hospitalizations and Emergency Department (ED) or Hospital Outpatient IV diuretic visits. This study retrospectively analyzed the two-year experience of 40 HF patients with NYHA class II or III regardless of the ejection fraction. All participants utilized the Cordella HF System to transmit daily data, including weight, blood pressure, heart rate, pulse-ox readings, and symptoms, via a wireless tablet. Cardiologists and nursing staff developed workflow procedures to enable daily monitoring. Recommendations for optimizing GDMT and lifestyle modifications were communicated to patients through telephone calls and tablet-based messaging. Patient compliance (data sent 5 out of 7 days) at 2 years was 95%, with clinical compliance being 100% (data review at least twice weekly). The frequency of phone calls resulting in medication adjustments surged six times with remote patient monitoring, whereas episodic office visits decreased by 14.5%. HF hospitalizations reduced by 83% at 2 years post enrolment [82]. CHFS is commercially available in the EU and US. 

These pilot studies highlight the benefits provided by remote monitoring in a small clinical setting and the potential to influence a broader HF population by facilitating comparable workflow patterns in a multi-center hospital setting.

### 3.2. Fluid Heart Tracker App

**Figure 10:**
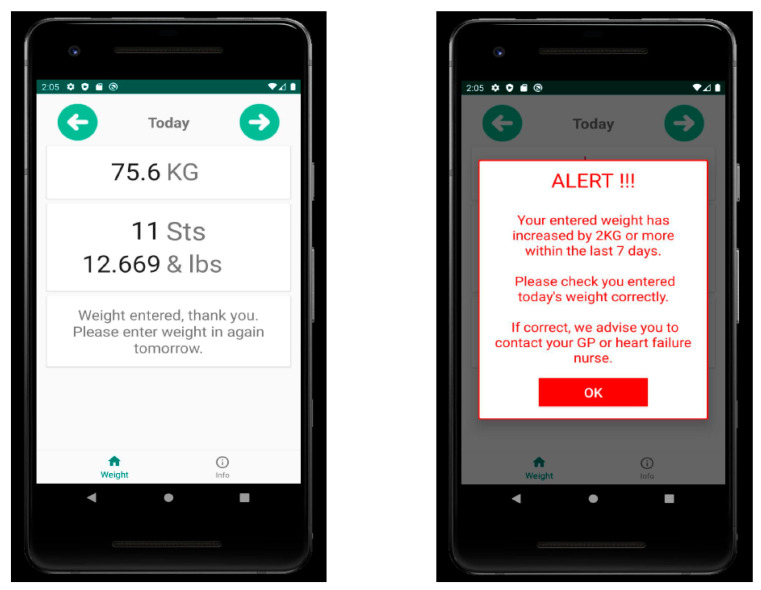
Example illustration of weight monitoring and clinic instructions and follow-up via the Fluid Heart Tracker Application. Used with permission from Caples N, The Fluid Heart Tracker App-knowing when to make contact for early medical intervention for deteriorating heart failure. Health Summit 2023 [86].

Fluid Heart Tracker App is a simple mobile application designed to log weight and notify the user of notable weight increases. The application stands out for its simplicity. Developed in collaboration with individuals living with HF (HF), its purpose is to prompt patients to seek medical assistance when their weight is on the rise. This app was developed by an advanced nurse practitioner at the University Hospital Waterford, Ireland (Figure 10). The rationale for this initiative is the persistence of chronic HF conditions in Ireland over the past few decades and the subsequent risk factors that impose a clinical and economic burden on Irish healthcare institutions. In Ireland, approximately 90,000 individuals are currently living with HF, with an additional 160,000 at risk. Fluid retention, evident through weight gain, serves as an indicator of HF. Prompt identification of weight gain prior to the onset of symptoms, along with timely intervention, is crucial for enhancing patient outcomes and minimizing associated healthcare expenses [85]. 

In Ireland, patients with HF are encouraged to record weights daily as part of routine care. Internationally, a weight gain of 2 KG or more over a 7-day period is recognized as a sign of deterioration, prompting the need for intervention. However, about 66% of HF patients experience mild cognitive impairment, affecting their ability to recognize weight increases. Helping patients identify weight gain could prompt them to seek assistance earlier, resulting in quicker intervention and better clinical results [85]. 

In a pilot study involving 31 HF patients with class II-III, the acceptability and usefulness of the Fluid Heart Tracker App were determined in three Irish tertiary hospitals. All patients who utilized the App found it beneficial and user-friendly and would recommend it to fellow HF patients. Currently, a larger national study is underway, which aims to evaluate the impact, effect, and perceived value of the Fluid Heart Tracker App on patients’ outcomes and service providers [86]. This study may shed some light on the impact of weight as the sole factor in optimizing therapy and addressing HF management.

### 3.3. Scale-HF 1 Study

A prospective, multi-center study SCALE-HF 1 (Surveillance and Alert-Based Multiparameter Monitoring to Reduce Worsening HF Events) is aimed aim at creating and evaluating the accuracy of the heart function index. This index is a comprehensive algorithm incorporating non-invasive hemodynamic biomarkers obtained from a cardiac scale, with the goal of predicting occurrences of deteriorating HF. HF patients are often advised to monitor their HF status by standing weight measurements. Through the Scale-HF 1 study, the idea of a novel weight scale to track multiple hemodynamic parameters will be assessed [87]. 

This study details only the design of Scale-HF 1 with a few patient examples highlighting the potential use of this cardiac scale as a comprehensive tool for monitoring cardiovascular hemodynamic status. This observational study aims to enroll around 300 patients with chronic HF who have recently experienced decompensation. Participants will be encouraged to take daily measurements using a cardiac scale. 

**Figure 11:**
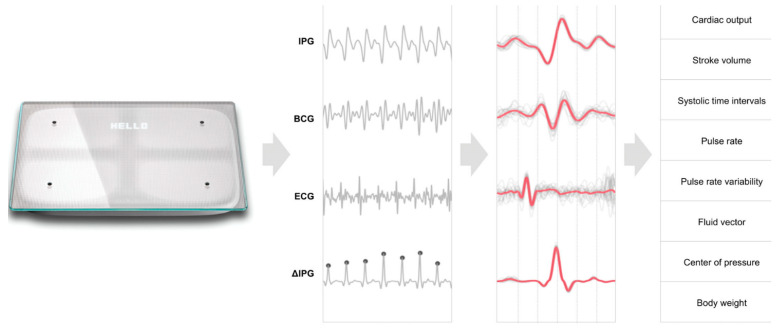
Representation of Bodyport Cardiac Scale and a list of parameters measured (BCG: Ballistocardiography; IPG: Impedance plethysmography) [87], Creative Commons CC BY License.

Approximately 50 HF events, characterized by emergency department visits, urgent or unscheduled appointments to clinics, or hospitalizations due to deteriorating HF, will be used for the development of the study model. The composite index will comprise hemodynamic biomarkers extracted from signals such as ECG, ballistocardiogram (BCG), and impedance plethysmogram measured by the cardiac scale (Figure 11). The biomarkers comprise weight, pulse rate variability, peripheral impedance, as well as estimations of stroke volume, cardiac output, and blood pressure. Parameters such as the sensitivity of the composite index, unexplained alert rate, and time required to predict worsening HF events will be evaluated and compared with conventional weight-based algorithms typically used in a clinical setting. Future studies will focus on validating the composite index and determining its potential to improve the outcome in HF patients [87].

The study is designed to include HF patients with both reduced and preserved ejection fractions, with few exclusion criteria, aiming for applicability across a wide HF population. The cardiac scale leverages the existing patterns among HF patients of daily weight measurements, reducing the potential resistance associated with adopting new technology. Clinical implications suggest that the heart function index may offer precise, early predictions of HF exacerbations without necessitating implantable or wearable devices. Subsequent research will validate this index and explore its capacity to facilitate timely interventions, potentially enhancing outcomes for HF patients [87].

### 3.4. Mobile Phone-Based Telemonitoring

**Figure 12:**
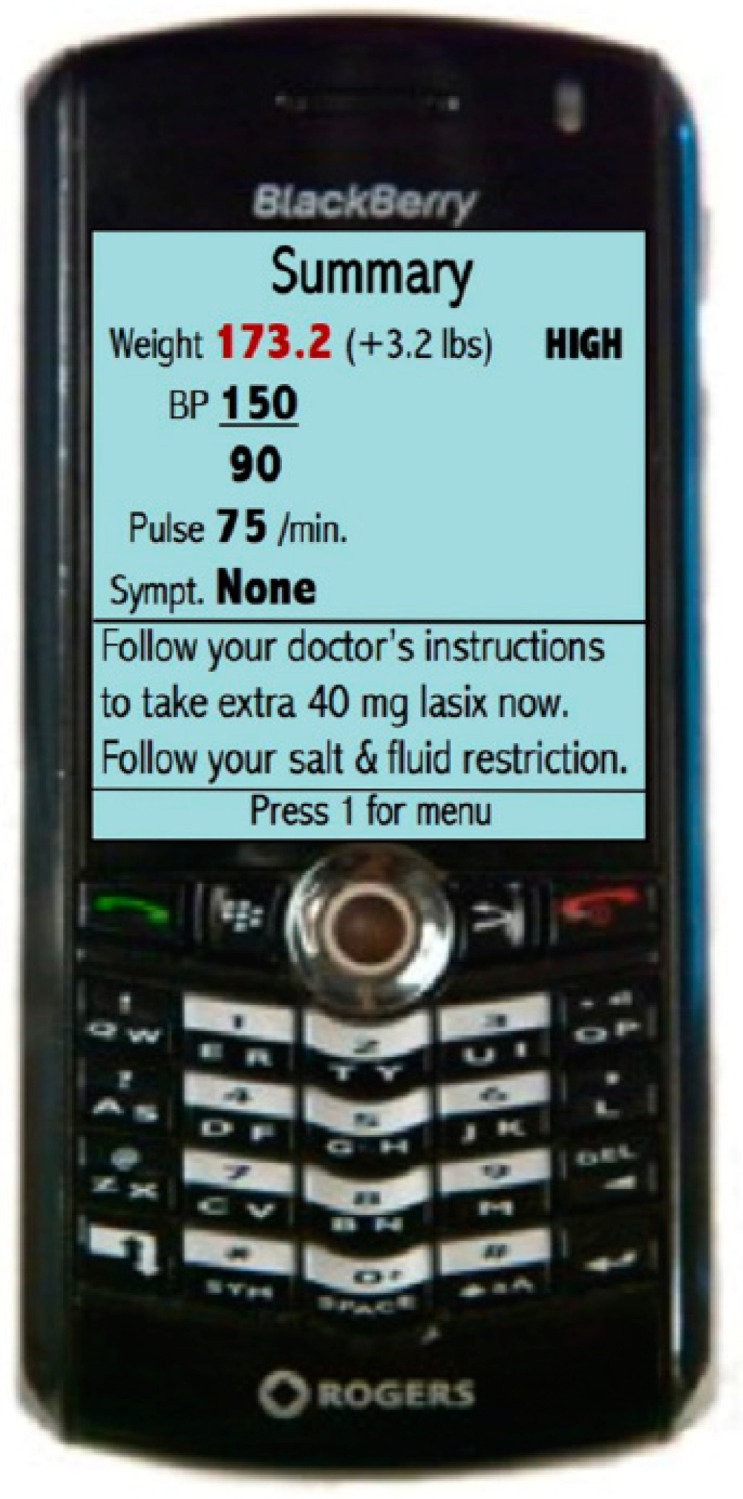
Demonstration of clinical response and follow-up through mobile phone-based monitoring [88], Creative Commons, CC BY License.

While mobile phone technology is increasingly prevalent and cost-effective, the feasibility and effectiveness of a telemonitoring system based on mobile phones have not been established widely. A randomized controlled trial involving 100 patients recruited from an HF Clinic in Toronto, Canada, investigated the impact of mobile phone-based telemonitoring systems on HF management and outcomes [88]. Patients were randomly allocated to either a telemonitoring group or a control group. Over a span of six months, the telemonitoring group (*n* = 50) conducted daily weight and blood pressure measurements, along with weekly single-lead ECGs. Additionally, they responded to daily health (symptom-related) questions via a mobile phone. These readings were wirelessly transmitted to the mobile phone and subsequently to data servers. Patients received instructions on their mobile phones, while the cardiologists received alerts when needed (Figure 12). 

This study demonstrated positive outcomes in the telemonitoring group with respect to quality of life (Minnesota Health Questionnaire; *p* < 0.05), self-care maintenance (Self-Care of HF Index; *p* = 0.03), decrease in Brain Natriuretic Peptide (BNP) levels (*p* = 0.02), improvement in left ventricular ejection fraction (*p* = 0.005). No significant differences were observed in the control group. Also, the control and telemonitoring group showed no significant differences in outcomes such as hospitalization, mortality, or emergency department visits due to a small sample size. However, utilization of this system demonstrated high compliance and feasibility among patients, including the older population and those unfamiliar with using mobile phone technology [88]. 

Another cross-sectional study was conducted in Canada to evaluate the safety, efficacy, and impact of two different platforms for remote monitoring of COVID-19 patients in maintaining quality care and patient engagement. The study also aimed to assess the acceptability, usability, and ease of use of these two platforms from the patient’s perspective. The first platform was known as Telecare-COVID and focused on telephone calls only, while the second one was a telemonitoring application known as CareSimple-COVID. A total of fifty-one patients participated in the study, with 18 patients utilizing the CareSimple-COVID platform and 33 patients utilizing the Telecare-COVID platform. Overall, both platforms achieved an 80% satisfaction rate regarding the quality and safety of services. Additionally, more than 88% of patients on each platform expressed satisfaction with the services, describing them as engaging, beneficial, easy to use, and tailored to their specific needs. Particularly, a survey brought to light significant differences in patient perspectives between the two platforms. Patients reported higher levels of empathy from their caregivers and perceived a higher standard of care quality and safety on the CareSimple-COVID platform compared with the Telecare-COVID platform. Patients highly appreciated features such as easy access to services and care team members, the user-friendly nature of the platform, continuity in care, and a range of services available. Nonetheless, they also identified technical limitations and expressed concerns about the importance of maintaining face-to-face interaction, data security, and confidentiality. Recommendations for improvement included promoting access to interconnected devices, improving communication regarding confidentiality protocols, and involving patients in the development and implementation of telehealth platforms. [89]. A combination of both platforms may be a viable option for implementation in a post-pandemic era and for other post-hospitalization populations. To optimize effectiveness, it is crucial to address the areas identified for improvement and the issues raised with a patient-centric approach [88,89]. 

The results of both studies mentioned above highlight the benefits of non-invasive telemonitoring systems in improving HF patient outcomes. However, we might still be decades away from realizing the true potential of such systems in a large sample size with an appropriate HF population. 

## 4. Conclusions and Future Outlook on Remote HF Management

The future of healthcare is poised for significant advancements with the integration of sensor technologies and telehealth solutions. Sensor technologies such as the CardioMEMS, Cordella, IHM, and LAP sensors offer unprecedented opportunities for real-time monitoring of cardiac parameters, enabling early detection and intervention of chronic HF. Additionally, the remote monitoring of CVP and IVC areas provides valuable insights into cardiac function and volume status, further enhancing patient care. Non-invasive telehealth solutions, including mobile apps and telephone interventions, hold immense potential in extending healthcare access and promoting patient engagement. As these technologies continue to evolve, the scope for personalized, proactive, and patient-centric care delivery expands, promising a future where healthcare is more accessible, efficient, and effective.

In addition, there is a growing trend for a hybrid approach to HF management that combines remote monitoring with traditional in-person care. This hybrid model allows for the best of both worlds, with patients benefitting from the convenience of remote monitoring while still having access to face-to-face interactions with their healthcare providers when necessary. From a future perspective, there is immense potential for multiparameter HF management using non-invasive telehealth platforms. Advances in technology, such as wearable devices and artificial intelligence (AI), are making it possible to monitor a wide range of parameters beyond just symptoms, including vital signs, biomarkers, and even physiological data. By incorporating these additional parameters into remote monitoring platforms, healthcare providers can gain a more comprehensive understanding of each patient’s condition and tailor their treatment plans accordingly [90]. 

From a clinician’s perspective, hemodynamic monitoring signifies the forthcoming frontier in HF treatment [79].

- Digital health offers the potential for more personalized management of HF by enabling continuous monitoring for both patients and physicians.
- Digital solutions, including those leveraging data from implantable devices, will aid in managing HF effectively.
- These tools are intended for patient use and warrant thorough investigation.
- Emerging clinical workflows, such as remote management and decision-making by utilizing data from telehealth platforms, demand further research and development [79].

The COVID-19 pandemic catalyzed a surge in TH utilization, a trend likely to persist beyond the pandemic’s resolution. Traditional face-to-face medical interventions are gradually transitioning to virtual platforms through TH, offering improved healthcare access and mitigating geographic disparities. TH also fosters efficiency gains within healthcare systems and enhances patient self-management and empowerment. By complementing human intervention, AI can further enhance TH, particularly in managing the complexities of comorbidities in HF, paving the way for a personalized approach to HF patient care. AI-powered TM with a multidisciplinary integrated care approach could represent a potential evolution in HF management [90].

AI plays a crucial role in predicting and preventing HF by analyzing biological markers and monitoring data [91,92]. Through advanced learning algorithms, machine learning techniques identify patterns in new data, improving disease prognosis and treatment decisions over time. Additionally, AI can combine data from various sources, facilitating the development of a single electronic patient record system and enabling a multidisciplinary approach to personalized medicine. Moving forward, HF patient data will be analyzed alongside information from various organs and systems, allowing for a more holistic patient-centric approach. Patient data generated from telehealth platforms and communications will be used to develop AI-driven models of disease progression [91,92].

From a patient’s perspective, the integration of remote monitoring technologies for managing HF has been shown to impact patient experience and satisfaction positively. Many patients express feeling well-informed about remote monitoring, with a significant portion indicating improved coping since its incorporation. Technical issues, while reported by some, have not been a widespread concern. Patients observed a noticeable extension in the intervals between in-person follow-up visits, enhancing convenience. Moreover, a substantial portion of patients show interest in smartphone-based data transfer, signaling a desire for streamlined connectivity. However, alongside this enthusiasm, concerns regarding smartphone-based data transmission linger, primarily revolving around data safety, battery consumption, memory capacity, and data usage. Despite these reservations, the overall patient experience with remote monitoring appears to be positive, with many appreciating the benefits it brings to their HF management journey. Both patient and physician adherence are essential for the success of remote monitoring technologies, as their effectiveness relies on proactive actions by healthcare providers guided by patient-derived data [89,93].

Overall, both the invasive and non-invasive TH platforms represent a promising avenue for improving HF management and outcomes. By promoting patient adherence and enabling more personalized and proactive care, these platforms have the potential to revolutionize HF management in the future.

## Figures and Tables

**Table 1:** Summary of sensor technologies describing the device, target location, parameters measured, indications, and their clinical evidence.

Device	Target Location	Parameters Measured	Indications	No. of Clinical Trials	No. of Pre-Clinical Trials	Data Collection
Titan^TM^ pressure sensor (ISS Inc., Ypsilanti, MI, USA) [38,39]	Left atrium	Left atrial pressure	NYHA class IV Patients with ICD/CRT-D Cardiomyopathy with optimal medical therapy	Two Feasibility and safety study (*n* = 40). Functioning of the sensor in patients with left ventricular assist device (*n* = 4).	>60 animal studies tested the sensor in all four chambers of the heart, as well as the aortic and pulmonary arteries.	External reader device that captures pressure signals and stores them on a computer that transmits data to a central server.
V-LAP^TM^ (Vectorious Medical Technologies), HeartPOD (St. Jude Medical, Minneapolis, MN, USA) [33,36,43]	Interatrial septum	Left atrial pressure, atrial ECG, body temperature	NYHA class III HFH in the last 12 months.	Three LAPTOP-HF (HeartPOD, *n* = 486) HOMEOSTASIS trial (HeartPOD, *n* = 40) VECTOR-HF trial (*n* = 30).	NA	External reader device (wearable belt) that transmits data to a secure cloud server.
Chronicle IHM (Medtronic, Inc., Minneapolis, MN, USA) [45,47,48]	Upper chest with a sensor attached to the right ventricle	Right ventricular pressure	NYHA class III-IV Compatible with single-chamber ICDs only	COMPASS-HF (*n* = 274).	NA	Pressure data is captured by the monitor’s sensing circuitry, stored in the memory, and transmitted via radiofrequency-linked telemetry to a programmer in the clinic.
CardioMEMS (Abbott) [54,57,58,64,65,68]	Pulmonary artery	Pulmonary artery pressure	FDA approved for NYHA class II-III HFH in the last 12 months Elevated BNP/NT-proBNP levels	Six CHAMPION (*n* = 550) GUIDE-HF (*n* = 1000) MEMS-HF (*n* = 234) COAST (*n* = 130) HEMOVADS (*n* = 10) MONITOR-HF (25 centres in Netherlands).	NA	External reader device (pillow) that transmits data to a secure cloud server.
Cordella (Endotronix, Inc. Lisle, IL, USA) [69,70,71]	Right pulmonary artery	Pulmonary artery pressure	NYHA class III HF HFH in the last 12 months FDA approval in process	SIRONA (first-in-human) (*n* = 15) SIRONA II (*n* = 70) PROACTIVE-HF	NA	External reader device (handheld monitor) that transmits data to a secure cloud server.
Fire1 (Foundry Innovation and Research 1 Ltd., Dublin, Ireland) [72,73]	Inferior vena cava	Inferior vena cava area and fluid volume	HFH or urgent HF visit in the last 12 months Elevated BNP/NT-proBNP levels On higher doses of loop diuretics (furosemide)	One FUTURE-HF (safety and efficacy study). (results pending)	Two Feasibility of implantation (*n* = 9). Safety and performance of the sensor under acute and chronic intravascular volume modulation (*n* = 20).	External reader device (belt) that transmits data to a secure cloud server.
Central venous pressure sensor (University of Galway, Ireland—Endotronix Inc., Lisle, IL, USA) [76]	Inferior vena cava	Central venous pressure	NYHA class III Recurrent admissions due to congestive HF Patients developing fluid retention/edema.	NA	One Feasibility and efficacy (acute) study (*n* = 2)	External reader device (handheld monitor) that transmits data to a secure cloud server.
Vascular electronic system/Wireless stent [81]	Right iliac artery	Arterial pressure, pulse rate, and flow	Coronary artery disease	NA	One Feasibility study (*n* = 1) showing minimally invasive catheter implantation of the smart stent in narrow arteries.	NA

Abbreviations—BNP: Brain Natriuretic Peptide, CRT-D: Cardiac Resynchronization Therapy with Defibrillator, FDA: Food and Drug Administration, HFH: HF Hospitalization, ICD: Implantable Cardioverter Defibrillator, NYHA: New York Heart Association.

**Table 2:** Summary of non-invasive telehealth technologies describing the technology used, parameters measured, indications, nature of study conducted, and patient access to their own metrics.

Study	Technology Used	Parameters Measured	Indications	Study Specifics (Pilot/Prospective/Retrospective/Observational/Experimental)	Patient Access
TIM-HF2 [23,24]	Smart-phone/ Application integrated with a secure cloud server	ECG, BP, SPO2, weight	NYHA class II-III HFH in the last 12 months LVEF ≤ 45%	Prospective, randomized multicentre trial	Yes
CHFS Ireland, CHFS US [82,83,84]	Smart-phone/ Application integrated with a secure cloud server	BP, HR, SPO2, weight	NYHA class I, II, III. NYHA Class II-III. HFH in the last 12 months HFrEF/HFpEF.	Prospective, single-center, observational study (Galway, Ireland), Retrospective multi-center study (Seguin, San Antonio, TX, USA).	Yes
Fluid Heart Tracker [85,86]	Smart-phone/ Application	Weight	NYHA class II-III Fluid retention Hospital readmissions Rapid weight fluctuations	Pilot, observational study	Yes
Scale-HF [87]	Smart scale integrated with smart phone-application (patient-end) and a clinical dashboard	ECG, BCG, IPG	HFrEF/HFpEF HFH in the last 12 months Fluid retention Elevated BNP/NT-proBNP levels Radiological evidence of pulmonary congestion Elevated right/left filling pressures (includes patients with implants).	Prospective, multicentre study	Restricted to weight, pulse rate, and fluid status only.
Mobile phone-based telemonitoring [88]	Mobile phone-based messaging (not app-based)	ECG (weekly), weight and BP (daily)	NYHA class II, III, IV Elevated BNP/NT-proBNP levels LVEF < 40% Expected survival of greater than one year.	Single-center, randomized controlled trial	Yes
Telecare and CareSimple-COVID [89]	Phone calls, Smart-phone/ Application	COVID-19 progression, medications, and follow-ups	COVID-19 disease. Additional infections (respiratory/COPD).	Single-center, cross-sectional study	No, Yes

Abbreviations—BCG: Ballistography, BNP: Brain Natriuretic Peptide, BP: Blood Pressure, COPD: Chronic Obstructive Pulmonary Disease, ECG: Electrocardiography, HFrEF/HFpEF: HF Reduced/Preserved Ejection Fraction, HFH: HF Hospitalization, IPG: Impedance Plethysmography, LVEF: Left Ventricular Ejection Fraction, NYHA: New York Heart Association, SPO2: Oxygen Saturation.
